# Retraction: Jaeumganghwa-Tang Induces Apoptosis via the Mitochondrial Pathway and *Lactobacillus* Fermentation Enhances Its Anti-Cancer Activity in HT1080 Human Fibrosarcoma Cells

**DOI:** 10.1371/journal.pone.0341705

**Published:** 2026-01-28

**Authors:** The PLOS One Editors

Following the publication of this article [1], concerns were raised about results presented in Figs 1–3, 5-6, S1, S4, and the statistical methodology reported in this article. Specifically,

- The following panels appear to partially overlap:
  - Fig 1A 0 JGT (µg/mL) and Fig 6A 0 JGT (µg/mL).
  - Fig 1B 0 JGT (µg/mL) and Fig 1B 1000 JGT (µg/mL).
  - Fig 5B 0 JGT (µg/mL) PD98059 and Fig 5B 0 JGT (µg/mL) SP600125.
  - Fig S1 AGS 0 JGT (µg/mL), 500 JGT (µg/mL), and 1000 JGT (µg/mL), and Fig S4 AGS 0 JGT (µg/mL), 500 JGT (µg/mL), and 1000 JGT (µg/mL) respectively.
  - Fig 6C aJGT t-ERK lanes 3-4 and Fig 6C fJGT162 t-ERK lanes 1-2.
- The Fig 2B tubulin and the Fig 3A tubulin panels appear similar.
- Multiple panels in Fig 6B appear to present vertical irregularities suggestive of splice lines. The assumed splice lines do not appear to be present consistently across all panels, raising concerns about the reliability of the associated density quantification results.
- The Materials and Methods section only reports the use of Student’s t-test and does not clarify the statistical approach used for multivariate analysis, whereas this article appears to present multivariate results.

In light of the unresolved concerns with Figs 1, 5, and 6, which call into question the validity and reliability of these results, the *PLOS One* Editors retract this article.

All authors either did not respond directly or could not be reached.

## References

[1] Kim A, Im M, Hwang Y-H, Yang HJ, Ma JY. RETRACTED: Jaeumganghwa-Tang Induces Apoptosis via the Mitochondrial Pathway and *Lactobacillus* Fermentation Enhances Its Anti-Cancer Activity in HT1080 Human Fibrosarcoma Cells. PLoS One. 2015;10(5):e0127898. doi: 10.1371/journal.pone.0127898 26020238 PMC4447448

